# Programmatically Localizing Diabetic Retinopathy Features in 45-Degree Retinal Photographs Using Anatomical Colocation

**DOI:** 10.3390/jcm13030807

**Published:** 2024-01-30

**Authors:** Timothy I. Murphy, Amanda G. Douglass, Peter van Wijngaarden, James A. Armitage

**Affiliations:** 1 School of Medicine (Optometry), Deakin University, Geelong, VIC 3216, Australia; murphyti@deakin.edu.au (T.I.M.); amanda.douglass@deakin.edu.au (A.G.D.); 2 Centre for Eye Research Australia, Royal Victorian Eye and Ear Hospital, Melbourne, VIC 3002, Australia; peterv@unimelb.edu.au; 3 Ophthalmology, Department of Surgery, University of Melbourne, East Melbourne, VIC 3002, Australia

**Keywords:** diabetic retinopathy, diabetes, fundus, location

## Abstract

**Background**: The aim in this study was to investigate the localization of diabetic retinopathy features at the posterior pole. **Methods**: This study extracted diabetic retinopathy feature locations from 757 macula-centered 45-degree fundus photographs in the publicly available DDR dataset. Arteriole and venule locations were also extracted from the RITE (n = 35) and IOSTAR (n = 29) datasets. Images were normalized to collocate optic disc and macula positions, and feature positions were collated to generate a frequency distribution matrix. Sørensen–Dice coefficients were calculated to compare the location of different features. **Results**: Arterioles occurred in two main, distinct arcuate patterns. Venules showed a more diffuse distribution. Microaneurysms were diffusely located around the posterior pole. Hemorrhages and exudates occurred more frequently at the temporal aspect of the macula. Cotton wool spots occurred in a region approximating the radial peripapillary capillaries. Intraretinal microvascular abnormalities and neovascularization were seen throughout the posterior pole, with neovascularization at the disc (n = 65) being more common than neovascularization elsewhere (n = 46). Venous beading occurred primarily between the first and third bifurcations of the venules. Diabetic retinopathy overall was more frequent in the temporal aspect of the macula. The location of cotton wool spots and exudates showed moderate similarity (0.52) when all data were considered, reducing to low similarity (0.18) when areas of low frequency were removed. **Conclusions**: Diabetic retinopathy occurs throughout the posterior pole but is more frequent in the temporal aspect of the macula. Understanding the location of diabetic retinopathy features may help inform visual search strategies for diabetic retinopathy screening.

## 1. Introduction

Diabetes mellitus is a global health concern, with 10.5% of the global adult population affected by the disease as of 2021 [1]. By 2045, it is expected that 783 million people will have diabetes [1]. Diabetic retinopathy (DR) is a common complication of the disease and is a leading cause of preventable blindness, with its prevalence positively correlated with the duration of the disease and glycosylated hemoglobin level [1,2]. It is recommended that people with diabetes be screened for retinopathy at least annually or biannually by an optometrist or ophthalmologist [3,4,5,6], depending on risk factors and local screening guidelines.

Diabetic retinopathy is graded on the basis of retinal features, with each grade corresponding to a risk of progression to proliferative disease [7]. Diabetic retinopathy presents initially with microaneurysms, after which a range of pathological features such as intraretinal hemorrhages, exudates, cotton wool spots, venous beading, intraretinal microvascular abnormalities (IRMAs), preretinal hemorrhages, and neovascularization manifest. When using the International Clinical Diabetic Retinopathy Disease Severity Scale [7], a grading system used widely by optometrists and ophthalmologists, severity is defined by the presence and extent of these features. The chance of developing proliferative disease, and therefore significant visual impairment or blindness, increases with severity [8]; hence, accurate grading affects management decisions and is imperative for reducing the risk of vision loss. The mismatch between the saliency of pathological features and disease severity for some of these features can provide a clinical challenge; the presence of IRMAs indicates at least severe nonproliferative diabetic retinopathy (NPDR), but it can, at times, have a subtle clinical appearance and, to the best of our current knowledge, may be present anywhere in the retina. Similarly, mild NPDR is differentiated from no retinopathy only by the presence of microaneurysms, which can be difficult to see in a fundus photo due to their size, shape, location, and irregular pigmentation of the surrounding retina [9]. This challenge was demonstrated by Sellahewa et al. [10], who showed poor agreement between optometrists when grading fundus photos showing no retinopathy (51.4%). Finally, the presence of a single intraretinal hemorrhage signifies moderate NPDR, and mischaracterizing this hemorrhage as a microaneurysm also affects grading accuracy.

Knowledge of the retinal locations at which features of diabetic retinopathy are most likely to be seen may serve as a guide for clinicians. It is well understood that diabetic retinopathy affects the retinal capillary networks, especially the radial peripapillary capillaries and deep capillary plexus [11]. The morphology of retinal capillary plexuses is nonuniform, with four layers in the peripapillary region reducing to three, two, and one in the more peripheral regions [12]. Hence, localized capillary nonperfusion impacts a larger area in the peripheral retina.

Unlike other features, cotton wool spots are known to occur more frequently in a restricted area. Cotton wool spots are an accumulation of cystoid bodies in the retinal nerve fiber layer, leading to fluid accumulation within the tissue [13,14]. The association between cotton wool spots and the radial peripapillary capillaries is well understood [14], with damage to these capillaries leading to cotton wool spot development.

Studies investigating feature localization in diabetic retinopathy have been undertaken, generally with the intention of assessing widefield or montage imaging methodologies. For example, the frequency of features in nine photographic fields covering 150 degrees (60%) of the retina has shown that microaneurysms, intraretinal hemorrhages, and exudates occur most frequently in the posterior pole, with other features seen most commonly in the posterior and nasal fields and rarely in the temporal fields [12]. Conversely, ultrawide field imaging has shown features to be predominantly temporal to the optic disc [15,16], and neovascularization has been found to occur most frequently in the supertemporal quadrant around the superior arcades [17]. Within the posterior pole, microaneurysms, hemorrhages, and exudates have been shown to appear more frequently in the left eye, clustering in the supertemporal region [18]. However, this finding is likely reflective of undersampling and low statistical power rather than having an anatomical or pathophysiological basis.

Despite these studies, no high-resolution frequency distribution exists for all diabetic retinopathy features. Such knowledge may assist in the early detection of disease and improve agreement between clinicians. This paper describes the distribution of diabetic retinopathy as seen in 757 fundus photographs from a Chinese population.

## 2. Materials and Methods

**Figure 1:**
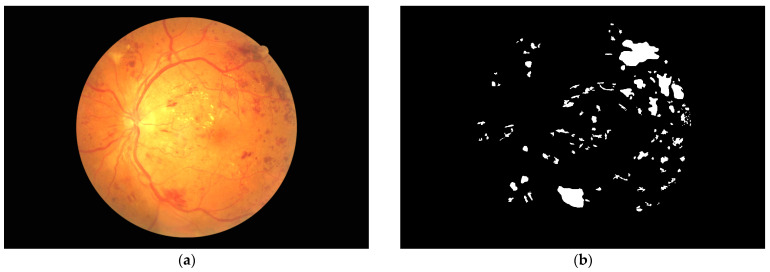
(**a**) Example fundus image; (**b**) corresponding hemorrhage annotation from the DDR dataset [19].

Deidentified retinal fundus photographs from the DDR dataset (no full term) [19] were obtained, together with binary ground truth maps corresponding to four diabetic retinopathy pathologies: microaneurysms, hemorrhages, exudates, and cotton wool spots, as demonstrated in Figure 1. This dataset contains 757 macula-centered images (371 left eye, 386 right eye) of various severity levels according to the International Clinical Diabetic Retinopathy Scale [7] (99 mild NPDR, 548 moderate NPDR, 34 severe NPDR, 74 PDR, 2 ungraded). To allow comparison of these feature locations to retinal landmarks, additional data were obtained from the Retinal Images Vessel Tree Extraction (RITE) [20] and IOSTAR (no full term) [21] datasets, containing fundus photographs and corresponding ground truth maps for arteriole and venule locations. One IOSTAR and five RITE images were excluded as they did not show both the macula and optic disc. The remaining images were collated (24 left eyes and 40 right eyes) and processed as a single dataset. IOSTAR and RITE data were only used to generate vascular maps.

Feature locations from different images could not be directly compared due to differences in centration, rotation, field of view, laterality, and image resolution. To overcome this, macula and optic disc colocation was performed. To do this, images were split into left and right eye groups, and, for each image in the group, the position of the optic disc and macula were manually recorded using custom software written by the authors (available at https://github.com/tim-murphy/dr_feature_localisation (accessed on 18 December 2023) version 1.0.0). Images were then scaled, rotated, and translated such that all maculae and optic discs were aligned, with the horizontal distance between the two arbitrarily set to 250 pixels and the macula positioned 25 pixels lower than the optic disc to reflect the disc–fovea angle [22]. The image canvas size was set to 1100 × 1100 pixels, as this was large enough to contain all normalized images. Corresponding ground truth maps underwent the same conversion and were stored as a 1100 × 1100 matrix, with 1 representing the presence of a given diabetic retinopathy or normal vascular feature, such as a microaneurysm or venule, and 0 representing the absence of a feature for that pixel location. Separate matrices were generated for each feature.

For each feature, corresponding ground truth matrices from all images were summed to create a frequency matrix for that feature, with each element representing the number of features found at that pixel location across all images. This process was performed separately for left and right eye data, with a combined matrix generated by mirroring the left matrix horizontally at the position of the macula and adding this to the right matrix. This process produced separate left, right, and combined frequency matrices for each feature. All matrices were then normalized so that element values ranged from 0 to 255. The data were then represented as heatmaps, with a value of 0 indicating no features at that location and 255 representing the location with the highest number of features.

The data were also split into groups based on disease severity. The DDR dataset includes severity grades for each image, with grading performed by seven ophthalmologists trained at institutions in Beijing who also earned a Certificate of Grading in Diabetic Retinopathy Screening from the Chinese version of the Gloucestershire Retinal Education Group [19]. Disease severity was based only on the fundus image, with no additional imaging or patient information available [19]. Each image was graded by four graders, with majority voting used to determine the final grade [19]. Where no consensus could be reached, a more experienced ophthalmologist made the final decision [19]. Additional information about the grading process was described by Li et al. [19].

As the original DDR dataset does not contain annotations for IRMAs, venous beading (VB), or neovascularization at the disc (NVD) or elsewhere (NVE), the authors created these annotations manually. Each image was assessed and labeled by three of the authors, who were registered optometrists with extensive experience in diabetic retinopathy grading (T.I.M., J.A.A., A.G.D.). Each reviewer individually annotated the four feature types present in each image using LabelImg software version 1.8.5 [23]. Annotations were collated and reviewed by the optometrists in the group for consensus. Where there was no unanimous agreement, a final decision was made by an ophthalmologist (P.vW.). Annotations were converted to frequency distribution heatmaps using the same process as above. Finally, a composite heatmap was created by summing heatmaps for all feature types, representing the frequency distribution for any diabetic retinopathy.

Several instances were noted where annotations contradicted the nominated grade, such as the presence of neovascularization in an image graded as nonproliferative diabetic retinopathy. These instances were documented by T.I.M, and changes to annotations and/or severity were agreed upon by J.A.A. and P.vW. A summary of these cases is included in Table 1.

Sørensen–Dice similarity coefficients [24,25] were used to compare the distribution frequency of different features. Where the frequency data showed region(s) of high concentration with sporadic presence elsewhere, the data were first trimmed to use only the most common locations. To do this, thresholding was used to create a cumulative frequency distribution, with the threshold best approximating the median cumulative frequency used as the threshold for data inclusion. This provided objective measures of absolute and trimmed similarities.

## 3. Results

**Figure 2:**
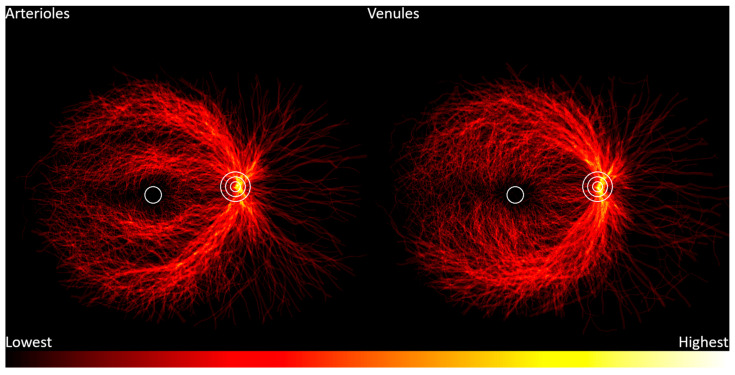
Frequency distribution of retinal vessels from lowest frequency (black) to highest frequency (white). Concentric circles represent the optic disc, and the single circle represents the macula.

Retinal vasculature location frequency heatmaps are shown in Figure 2. These heatmaps use data from the RITE and IOSTAR datasets. Arterioles are distributed in two main distinct arcuate patterns: the temporal arcades and a tighter band around the macula. The nasal vascular arcades are much less prominent. Venules show a more diffuse distribution.

**Figure 3:**
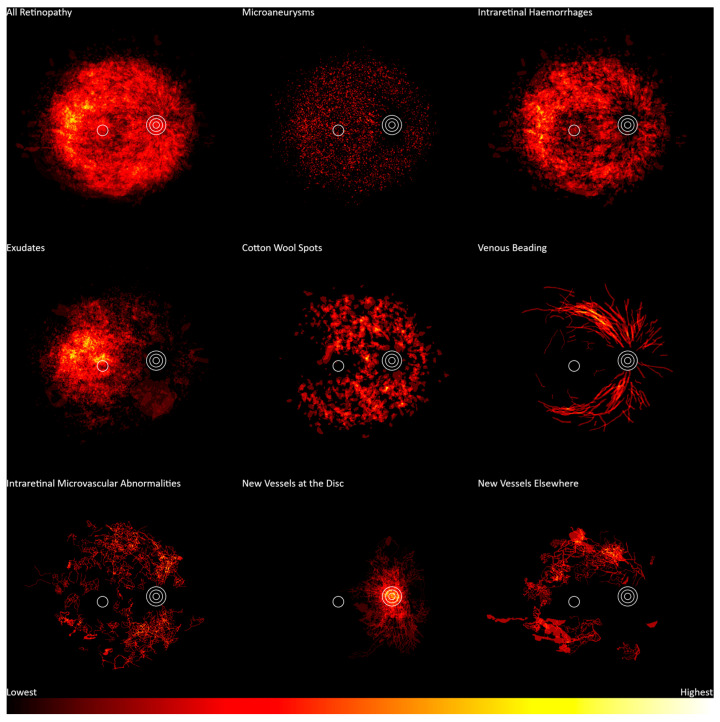
Frequency distribution of diabetic retinopathy features from lowest frequency (black) to highest frequency (white). Concentric circles represent the optic disc, and the single circle represents the macula.

Diabetic retinopathy location frequency heatmaps are shown in Figure 3. These heatmaps use data from the DDR dataset across all disease severities. Exudates (n = 486) occurred more frequently in the region including and immediately temporal to the fovea. Intraretinal hemorrhages (n = 601) occurred over most of the posterior pole, with a lower frequency around the center of the macula and a higher frequency temporal to the fovea. Microaneurysms (n = 570) showed a uniform distribution around the posterior pole. Cotton wool spots (n = 239) were dispersed in an arcuate distribution centered on the optic disc, facing the temporal retina, but sparing the region temporal to the fovea. Venous beading (n = 105) occurred principally in the proximal parts of the vascular arcades.

Neovascularization (n = 82) occurred most frequently at the optic disc (n = 65), with a lesser frequency around the arcades and temporal to the disc (n = 46). Intraretinal microvascular abnormalities (n = 74) had a relatively uniform distribution around the posterior pole but were not present at the optic disc or fovea. The number of samples for these two features may be inadequate to fully represent the distribution.

**Figure 4:**
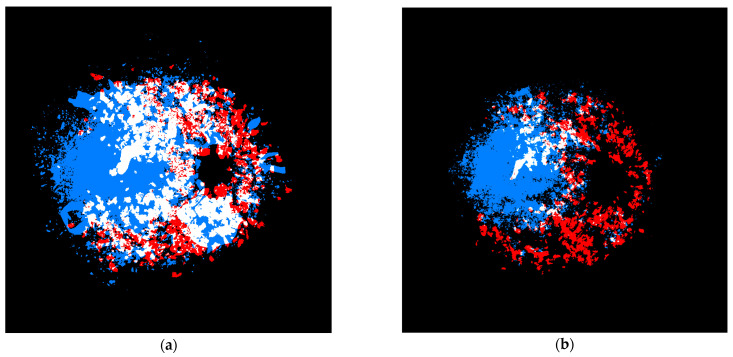
Location of exudates (blue) and cotton wool spots (red), with overlapping locations highlighted in white. (**a**) All data; (**b**) trimmed to include only the most frequent locations.

The distribution of exudates and cotton wool spots appeared to have little overlap. This finding has not been previously reported, so it was investigated further. To quantify the overlap, Sørensen–Dice coefficients were calculated twice: once using all annotated data and again where areas with few annotations were trimmed as described in the methods section. This resulted in two similarity coefficients: an absolute similarity of 0.52 and a trimmed similarity of 0.18. This indicated that for pixels where at least one exudate or cotton wool spot was located, there was a moderate degree of overlap between the two. However, when the pixels with fewer feature counts were removed, these features had little overlap. This is illustrated in Figure 4, with moderate overlap evident with all data and little overlap when considering only the most frequent locations.

**Figure 5:**
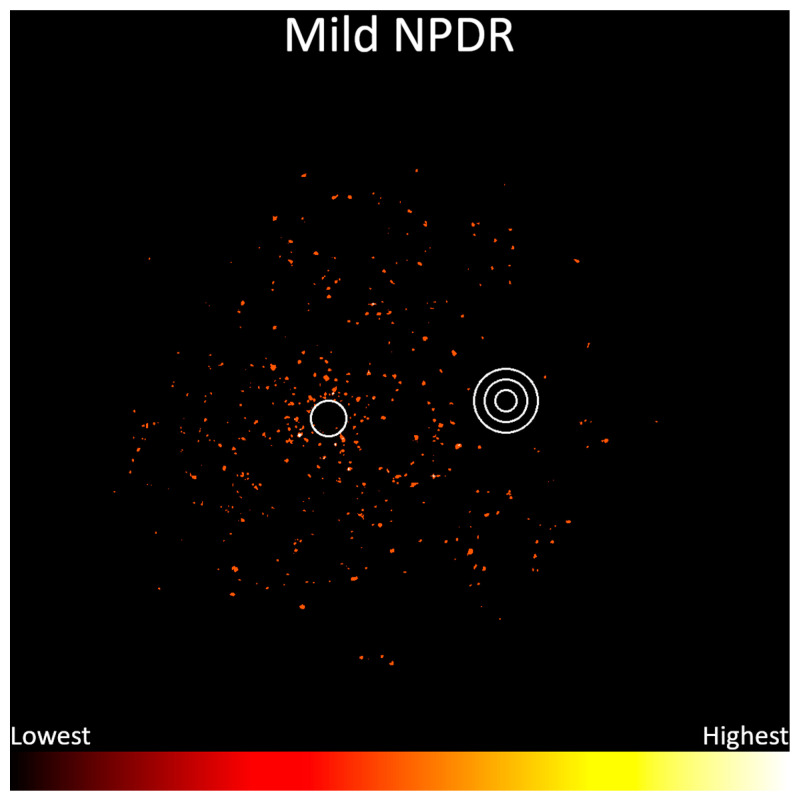
Frequency distribution of mild nonproliferative diabetic retinopathy, from lowest (black) to highest (white). Concentric circles represent the optic disc, and the single circle represents the macula.

Frequency data were then separated per disease severity according to the International Clinical Diabetic Retinopathy Severity Scale, and heatmaps were generated for each of these subsets. Mild nonproliferative diabetic retinopathy (n = 96), shown in Figure 5, demonstrates a diffuse distribution of microaneurysms around the posterior pole, with a higher frequency around the para- and perifoveal regions. This severity includes only microaneurysms by definition [7], so it is presented as a single heatmap.

**Figure 6:**
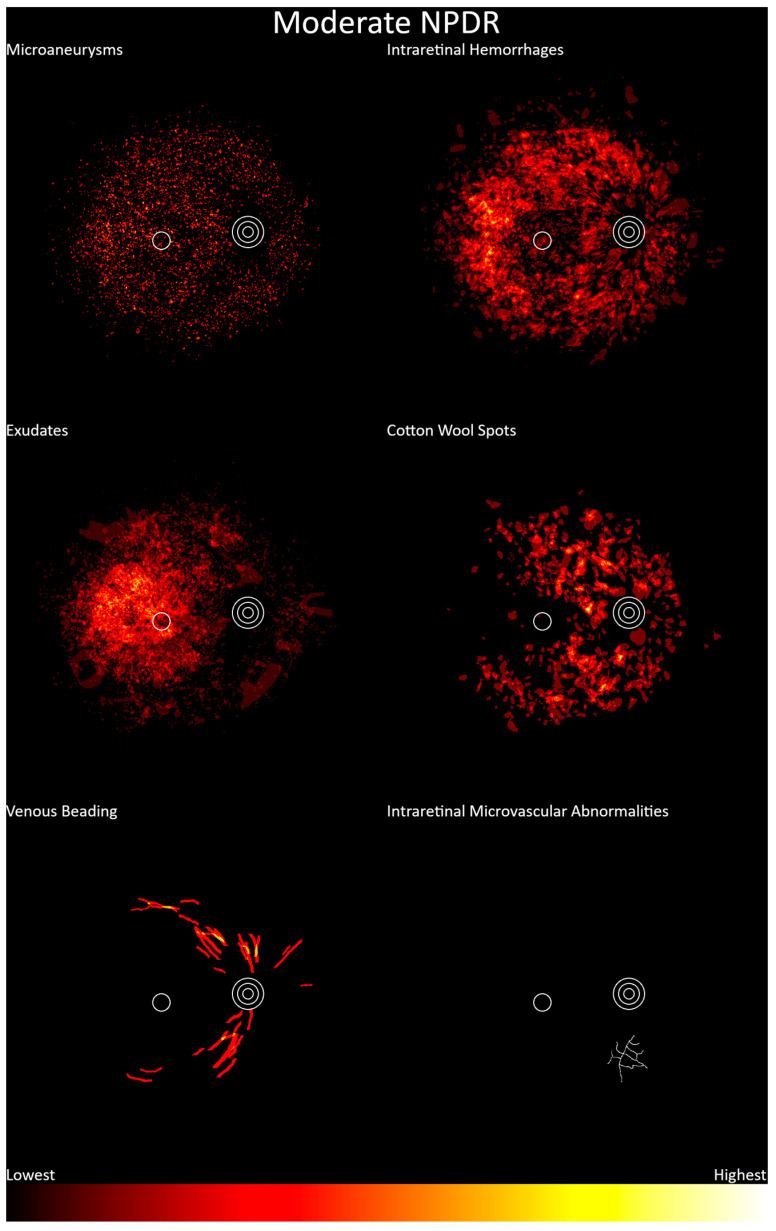
Frequency distribution of moderate nonproliferative diabetic retinopathy, from lowest (black) to highest (white). Concentric circles represent the optic disc, and the single circle represents the macula.

Moderate nonproliferative diabetic retinopathy heatmaps are shown in Figure 6 (n = 538). These show a higher probability of features in the temporal aspect of the macula, but features were present throughout the posterior pole. Microaneurysms, as seen in mild nonproliferative diabetic retinopathy, were diffusely located. Exudates occurred more frequently at the temporal aspect of the macula. Intraretinal hemorrhages were evident throughout the posterior pole with a higher frequency temporal to the fovea. Cotton wool spots were located throughout the posterior pole except at the fovea and temporal aspect of the macula. Venous beading was seen primarily between the first and third bifurcations of the retinal venules, though the data were insufficient to determine areas of high or low frequency. A single instance of IRMA was noted but was not considered sufficiently prominent to warrant a more severe grading.

**Figure 7:**
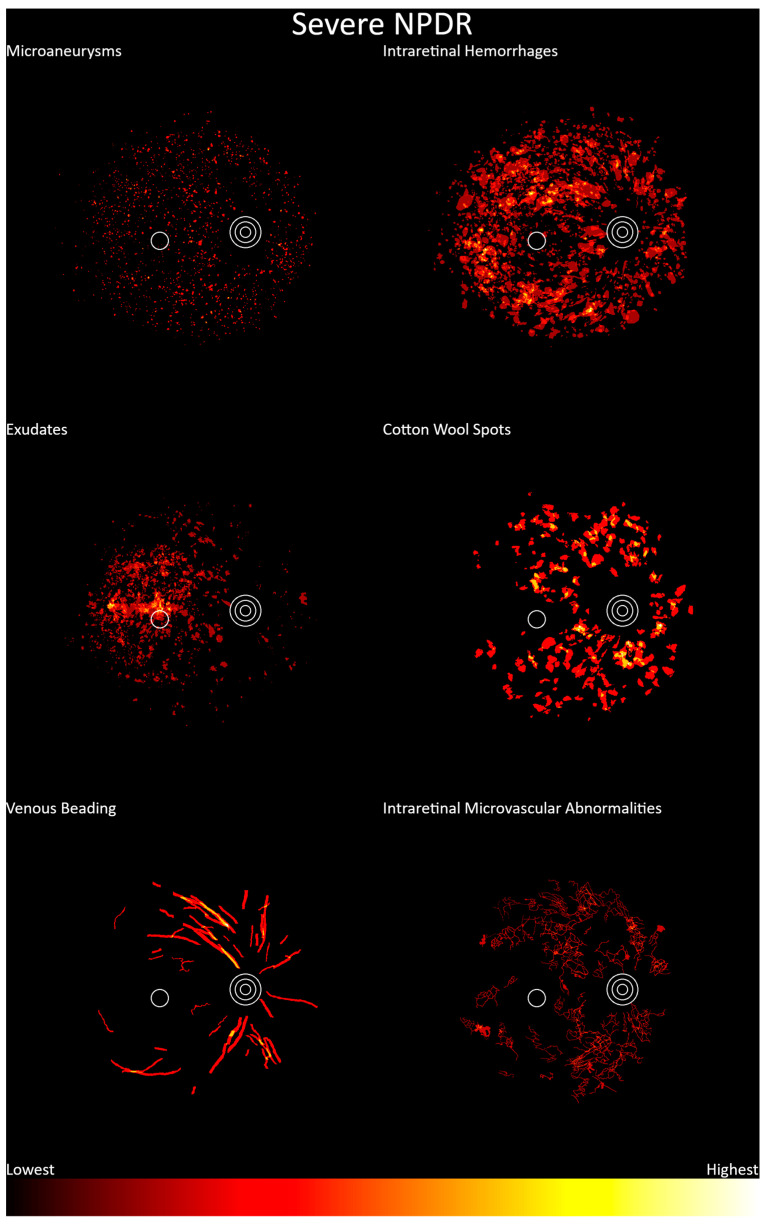
Frequency distribution of severe nonproliferative diabetic retinopathy, from lowest (black) to highest (white). Concentric circles represent the optic disc, and the single circle represents the macula.

Severe nonproliferative diabetic retinopathy heatmaps are shown in Figure 7 (n = 42). Features were apparent throughout the posterior pole. Microaneurysms, intraretinal hemorrhages, exudates, cotton wool spots, and venous beading showed similar distributions to those for photographs graded as moderate nonproliferative diabetic retinopathy. IRMA was seen more frequently nasally to the fovea, though this dataset consisted of only 32 annotations, and thus it was likely underpowered for this feature.

**Figure 8:**
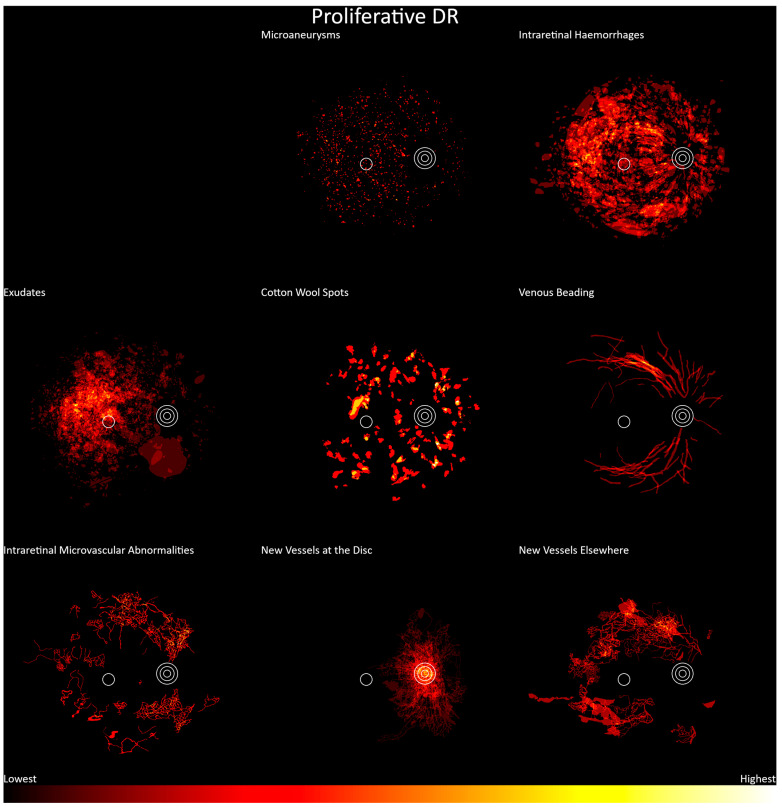
Frequency distribution of proliferative diabetic retinopathy, from lowest (black) to highest (white). Concentric circles represent the optic disc, and the single circle represents the macula.

Proliferative diabetic retinopathy is shown in Figure 8 (n = 81). Again, these show retinopathy features diffusely distributed around the posterior pole. Microaneurysms, intraretinal hemorrhages, exudates, and cotton wool spots showed the same pattern of distribution as moderate and severe nonproliferative diabetic retinopathy. IRMAs were seen around the posterior pole but were generally absent between the fovea and optic disc. Venous beading extended from the first to fourth bifurcations of the venules, a wider range than was seen in those with less severe retinopathy gradings. Neovascularization occurred mostly at, or adjacent to, the optic disc but was found elsewhere in the posterior pole, except at the fovea.

## 4. Discussion

This study investigated the location of diabetic retinopathy features and retinal vasculature at a high resolution in the central 45 degrees of the ocular fundus. Our findings show that diabetic retinopathy features are not evenly distributed throughout the posterior pole and we are the first to localize, at the pixel level, microaneurysms, intraretinal hemorrhages, cotton wool spots, exudates, intraretinal microvascular abnormalities, and neovascularization in the same study. We are also the first to use retinal colocation to allow feature locations to be compared programmatically.

In this analysis, the location of diabetic retinopathy features largely matches our expectations based on our understanding of the pathophysiology of the disease. It is understood that cotton wool spots result from capillary dropout in the radial peripapillary capillaries, leading to nerve fiber swelling [14]. These capillaries exist in a specific distribution around the optic disc [26], and we would thus expect cotton wool spot localization to approximate this area. Indeed, the location of cotton wool spots in this study closely approximates the distribution of retinal peripapillary capillaries identified in the work of Barbosa et al. [26].

This study suggests a low correlation between the distribution of cotton wool spots and exudates around the posterior pole, especially when considering areas where these features occur most frequently. Work by Munuera-Gifre et al. [18] suggests exudates are more frequent temporal to the fovea compared to nasally, which agrees with our findings. The location of cotton wool spots is also well understood, as described earlier. However, to the best of our knowledge, the low correlation between the two features has not been addressed. The radial peripapillary capillary region has an additional capillary plexus and therefore an increased vascular supply compared to the rest of the posterior pole. Using the location of the cotton wool spots as a surrogate for this capillary plexus, we see the region is relatively spared from exudation. The authors postulate that the additional vascular supply results in a lower susceptibility to exudation, leading to a higher prevalence in the temporal macular region where blood flow remains high [27] but vascular coverage is reduced. Further research in this area is warranted.

Microaneurysms occur frequently on the arteriolar side of capillaries in diabetes [28] and have been observed to occur more frequently temporal to the optic disc when glycemic control is moderate, becoming homogenous with poor control [29]. Our results show microaneurysms occurring diffusely around the posterior pole. As microaneurysms rupture, intraretinal hemorrhages, edema, and exudation develop [30]. Intraretinal hemorrhages occur around the posterior pole with an increased frequency temporal to the macula. This increased frequency may also result from reduced vascular supply in this region, though this requires further study.

Venous beading was most frequently observed on the larger venules of the superior and inferior arcades, primarily between the first and third bifurcations. This study did not indicate regions with a higher prevalence of IRMA and neovascularization elsewhere; however, further research is warranted due to the low number of data points for each of these features.

When analyzing features by severity, the frequency distribution patterns for each feature were similar between grades. As the dataset does not include patient metadata such as time since diagnosis, glycated hemoglobin levels, or comorbidities, this study was unable to determine which locations, if any, are the first to show signs of disease. We were also unable to determine if these frequency distributions differ in the presence of hypertension, dyslipidemia, or other common comorbidities. While datasets of retinal photographs with additional patient data exist, at the time of writing, none of these included feature annotations, and adding these annotations manually is a difficult task. Artificial intelligence systems are being developed to automate this process [31]. However, these systems cannot yet detect all features reliably and would still need to be checked by one or more expert graders. We believe advances in artificial intelligence technology will make this process easier in the future. Further studies considering these additional variables are recommended once this technology becomes available.

The feature localization data presented in this study may help clinicians understand outputs from artificial intelligence systems. Such systems often provide visual explanations of their output in the form of heatmaps [32,33,34]. Localization knowledge may provide insight into the features that are most influential in artificial intelligence system classifications.

The technique used for this analysis is simple and could be applied to other imaging modalities, including those in other healthcare sectors, where two common anatomical features can be used for image normalization. The authors have made the software available and encourage use by other research teams.

This study is not without limitations. The DDR dataset contains data sourced from Chinese hospitals; the IOSTAR data are from a private company in The Netherlands; and the RITE dataset uses data from DRIVE [35], which is sourced from a diabetic retinopathy screening program in the Netherlands. Though no data are available regarding the ethnicity characteristics of these datasets, we can assume these data approximate the ethnic profiles of the countries in which they were collected. While differences in retinal vessel geometries [36,37] and prevalence of retinopathy [38] have been demonstrated between ethnic groups, the authors do not expect our results to differ significantly from other ethnic populations. Replication using more diverse populations is warranted to test this assumption.

Our study also limited localization to the central 45 degrees of the retina, and retinopathy frequently occurs outside of this area [12,15,16]. Future work using widefield, ultra-widefield, or montaged imaging is warranted to identify areas of high susceptibility elsewhere in the retina.

The results of this study have not been verified in a clinical setting. While the data used for this study were sourced from public hospitals in China, they may not be representative of private ophthalmology clinics or other clinical settings. Such verification should be undertaken in various settings.

Overall, this work provides detailed frequency distributions for diabetic retinopathy features across all grades of severity, with specific patterns evident for exudates and cotton wool spots. These data may help inform a visual search strategy to improve accuracy and efficiency for eyecare providers screening for diabetic retinopathy.

## Figures and Tables

**Table 1:** Grade changes for DDR images.

Image	Old Grade	New Grade	Comments
007-2689-100.jpg	Mild NPDR	Moderate NPDR	Hemorrhages present
007-2697-100.jpg	Mild NPDR	Moderate NPDR	Hemorrhages present
007-2753-100.jpg	Moderate NPDR	Proliferative DR	NVD present
007-2788-100.jpg	Mild NPDR	Severe NPDR	Venous beading and hemorrhages present
007-3308-200.jpg	Moderate NPDR	Severe NPDR	Venous beading and hemorrhages present
007-3527-200.jpg	Moderate NPDR	Severe NPDR	Venous beading in two quadrants
007-3916-200.jpg	Moderate NPDR	Severe NPDR	Venous beading in two quadrants
007-3933-200.jpg	Moderate NPDR	Proliferative DR	NVD present
007-3975-200.jpg	Moderate NPDR	Severe NPDR	IRMA present
007-4268-200.jpg	Moderate NPDR	Proliferative DR	NVD present
007-4847-300.jpg	Severe NPDR	Proliferative DR	NVD present
007-5106-300.jpg	Moderate NPDR	Severe NPDR	IRMA present
007-5135-300.jpg	Moderate NPDR	Severe NPDR	IRMA present
007-5434-300.jpg	Moderate NPDR	Severe NPDR	IRMA present
007-5885-300.jpg	Severe NPDR	Proliferative DR	NVD present
20170505094751770.jpg	Moderate NPDR	Severe NPDR	IRMA present
20170518092011904.jpg	Moderate NPDR	Severe NPDR	IRMA present
20170602155248106.jpg	Severe NPDR	Proliferative DR	NVD present

## Data Availability

Publicly available datasets were used in this study. Acquisition instructions and software can be found here: https://github.com/tim-murphy/dr_feature_localisation (accessed on 18 December 2023).
